# Serotyping, a challenging approach for *Toxoplasma gondii* typing

**DOI:** 10.3389/fmed.2023.1111509

**Published:** 2023-04-06

**Authors:** Susana Sousa, Maria Fernandes, José Manuel Correia da Costa

**Affiliations:** ^1^Center for the Study of Animal Science (CECA), University of Porto, Porto, Portugal; ^2^Department of Infectious Diseases, R&D Unit, National Health Institute Dr. Ricardo Jorge (INSA), Porto, Portugal; ^3^Associate Laboratory for Animal and Veterinary Sciences (AL4AnimalS), University of Porto, Porto, Portugal; ^4^Associated Laboratory for Green Chemistry (LAQV) of the Network of Chemistry and Technology (REQUIMTE), Department of Chemistry and Biochemistry, Faculty of Sciences, University of Porto, Porto, Portugal; ^5^Center for the Study of Animal Science, University of Porto, Porto, Portugal

**Keywords:** serotype, *Toxoplasma gondii*, strain, antibodies, polymorphism, peptides

## Abstract

Genotype analysis has revealed a high genetic diversity in strains of *Toxoplasma gondii*, isolated from a wide range of intermediate hosts and different geographic origins. Diversity is notably striking for parasites from wild hosts in South America, generally referred as non-archetypal genotypes. Those genotypes are implicated in the etiology of severe clinical disease, multivisceral toxoplasmosis, associated with high rate of mortality in immunocompetent individuals. Can we accept specific antibodies produced during *T. gondii* infection as biomarkers to identify infecting genotypes? Scientific evidence supports a positive response to this question; however, the genetic diversity of *T. gondii* genotypes organized into 16 haplogroups and collectively defined in 6 major clades, provides a reminder of the complexity and difficulty for the purpose. This review discusses serological approaches to genotyping *T. gondii*.

## Graphical Abstract

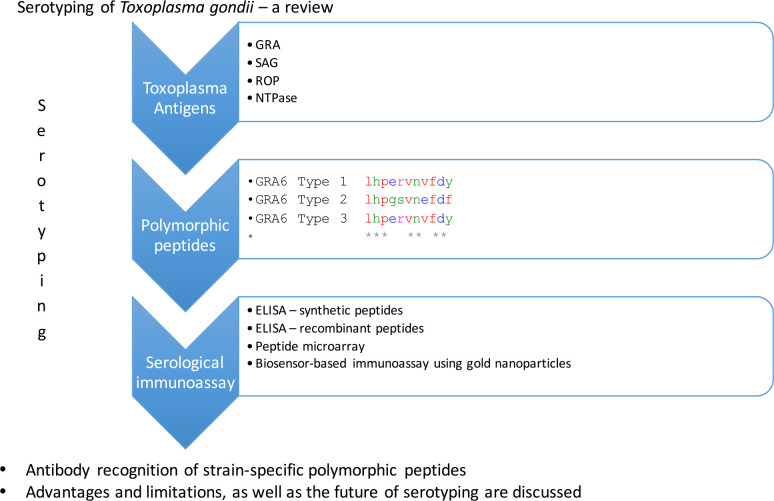

## 1. *Toxoplasma gondii* and toxoplasmosis, an overview

Toxoplasmosis is the most reported parasitic zoonosis in Europe. The global prevalence of toxoplasmosis is estimated to be around 30% with 10 million clinical cases (1–4) and it is ranked as the third most important contributor to health burden caused by food-borne illness in Europe (4). Most infections in humans are asymptomatic. However, severe complications may occur during (i) congenital *Toxoplasma* infection, such as abortion, stillbirth, and hydrocephalus in newborns (1, 4, 5); (ii) ocular toxoplasmosis, with retinochoroidal lesions leading to chronic ocular disease (1, 2); (iii) encephalitis in immunosuppressed patients (1, 4, 5); and (iv) multivisceral toxoplasmosis due to atypical genotypes in South America (6–8). A possible implication of *Toxoplasma gondii* genetic diversity on the pathogenesis of toxoplasmosis has been postulated (9–11). However, a better comprehension of the population structure is crucial in order to predict the potential spread of pathogenicity (or virulent) determinants related with isolates of *T. gondii*, their geographic and host origin. This process must be performed based on the study of a large population and representative of the *T. gondii* gene pool circulating in nature. Carme et al. (12) postulated a novel scenario in French Guiana wherein atypical genotypes of *T. gondii*, were associated to severe toxoplasmosis acquired by immunocompetent adults. The investigators claim that “…*this unusual form of toxoplasmosis is associated with an unusual genotype of T. gondii and may be linked to a neotropical forest based cycle*…” and that “*the “wild-type” strain of T. gondii should be isolated from neotropical mammals so that we can study its virulence and genetic characteristics*….” There is some consensus that the population structure of *Toxoplasma* includes the three archetypal genotypes (I, II, and III), along with non-archetypal genotypes. Archetypal strains, particularly genotype II and III are the most prevalent in Europe. In addition, in North America, along with type II and III, strains belonging to haplogroup 12 has been described in the sylvatic reservoir; finally, specific clonal strains have been described in Africa, South America, and Asia, whereas non-archetypal genotypes prevail in South America (11).

Can we anticipate that pathogenesis of toxoplasmosis may reflect the genotype structure of infecting strains? A new area of investigation has focused on this issue: a systematic isolation of parasites by bioassay of *T. gondii* of livestock, wild animals and humans (13). In addition, multilocus genotyping approach of *T. gondii* isolates provides data related with genetic diversity, including those genotypes favoring virulence (13). Both approaches are crucial to comprehend the mechanisms implicated in host derived genotype selection, and the pathogenesis of the disease. Both approaches are labor intensive and biological samples for bioassay are not always available. Moreover, the requirement for the parasite isolates and/or DNAs in bioassays may favor the isolation of one strain, while other may be missed in cases of co-infections with different strains.

In addition, to achieve genotype analysis, different approaches have been proposed: (i) PCR-RFLP (14–18); (ii) gene sequencing (multilocus genes sequencing and whole genome sequencing (19, 20)); and (iii) length polymorphism of microsatellite sequences (21–24). Table 1 summarizes the advantages and limitations of various genotyping methods.

**TABLE 1:** Advantages and limitations of genotyping methods.

	Advantages	Limitations	References
PCR-RFLP	Discriminative differentiation of archetypal strains	Time consuming; Parasite and/or DNA isolation	(18)
Microsatellites	High discriminative power for archetypal and non-archetypal strains; Detection of mixed infections	Time consuming; Parasite and/or DNA isolation	(23)
Sequencing	Detects all SNPs, deletions and insertions present in the gene	Time consuming; Parasite and/or DNA isolation	(19, 20)

Herein, serotyping is our focus, including on the state of the art, and controversies around effectiveness of approaches, considering the *spectrum* of *T. gondii* strains.

## 2. Serotyping: The other way of typing *Toxoplasma gondii*

The isolation of *T. gondii* from human samples is complex. Serotyping appears to be an alternative method for typing *T. gondii* strains. The process does not require isolation of the parasite. Serotyping is based on the antibody recognition of strain-specific polymorphic peptides (25–29). Early findings indicated that serotyping was informative and a promising approach. It requires access to sera from both symptomatic and asymptomatic humans. Serotyping limitations are related with the sensitivity and specificity of peptides investigated. The critical limitations are related with difficulties in differentiating the clonal genotype I and III, and all the non-archetypal genotypes (28, 29).

## 3. Methods to serotype *T*. *gondii*

Different strategies have been described to serotype *T. gondii*. Immunoenzymatic assays (ELISA) using polymorphic synthetic peptides are widely accepted (25, 28, 29); small peptides with a cysteine residue added to either the C or the N terminus of each peptide and a carrier protein such as keyhole limpet hemocyanin (KLH) (25), or synthetic peptides used by Sousa et al. (28) with a triple repeats of the polymorphic sequence in a single synthetic peptide. The use of synthetic peptides as ELISA antigens can be problematic due to the small size of the peptides. Such peptides are difficult to attach to the plate and consequently the sensitivity of the assay will be lower. To cross this problem and to facilitate peptide attachment to the plate and consequently improve ELISA sensitivity, small peptides can be coupled to carrier proteins (25). As an alternative, another approach was proposed and consisted on a trimer of the chosen peptide (if smaller than 20 aa) (28). This would increase the antigen size, with the objective of facilitating attachment to microplates and the antibody recognition. Peptides were coupled to the Immobilizer Amino plates (Nunc). These plates are specially adapted for small molecules, like peptides. The use of these plates replaces the role played by the carrier proteins. In another approach, polymorphic recombinant peptides expressed as polypeptides fused to *Escherichia coli* glutathione *S*-transferase or to a His-tag were employed as the target antigen in enzyme-linked immunosorbent assays (26, 30). The advantage is that bigger sequences, with more SNPs, are expressed for genotype I, II, and III. However, these recombinant polymorphic peptides discriminate genotype II, but cross-reactivity was found between genotype I and III. Peptide microarray assays has been assessed (27, 31–33). The major advantage of these assays is screening large number of peptides from different antigens. Furthermore, they provide higher sensitivity than ELISA-based assays (27, 30, 32). Similar conclusions were obtained comparing these two methods on other infectious diseases (34, 35).

Finally, a novel gold nanoparticles (AuNPs) assay improved the sensitivity for genotype II. This procedure uses AuNPs conjugated with a polymorphic synthetic peptide previously identified (25, 28), and presents values suggesting higher sensitivity when compared with the classical ELISA approach (36).

## 4. Antigens and peptides: Which are the best targets for serotyping

If we accept that specific antibodies elicited during toxoplasmosis are useful as biomarkers for *T. gondii* genotypes, a new question emerges: which are the best targets for serotyping? Polymorphic regions from native *Toxoplasma* antigens can be identified by multiple sequence alignments of genotype specific target antigens (25, 26, 29). Polymorphic peptides can be predicted using a bioinformatics-based approach, and synthesized, according to these criteria:

1. The content of charged and/or hydrophilic amino acids,
2. a predicted α-helical structure,
3. presence of proline residues;
4. the presence of short regions of typically 8–12 aa that are bounded by 2 cysteine residues (25, 32);
5. high antigenic index, and
6. high surface probability (32).

Different selected peptides derived from a variety of *Toxoplasma* antigens, such as granule dense proteins (GRA), surface antigens (SAG), rhoptry proteins (ROP), and NTPases, have been explored (25, 26, 29, 32, 33, 37). Table 2 provides global data. Curiously, a limited number of peptides were able to differentiate *T. gondii* serotypes, and we assumed that polymorphic site and length of peptides might play a pivotal role in serological typing (38).

**TABLE 2:** Antigens from which polymorphic peptides were selected and used for serotyping.

Antigen	References
GRA1, GRA3, GRA4, GRA6, GRA7, NTPase-I and III, SAG1, SAG2, SAG3, SAG4, BSR4, SRS2, ROP1	(25)
GRA5, GRA6, GRA7, SAG2A	(32)
GRA5, GRA6	(26)
GRA6, GRA7	(29)
GRA9, GRA31, ROP9, ROP10, ROP16, ROP 19A, ROP20, ROP25, ROP26, ROP39, ROP47	(33)

Peptides derived from native SAG2, GRA3, GRA6, and GRA7 antigens proved ability to differentiate *Toxoplasma* strains in infected mice (25). However, these findings are not reproduced, completely, in humans. Meanwhile, GRA6 and GRA7 (Table 3) derived peptides evidences ability to differentiate genotype II from non-genotype II strains in human sera (25). The presence of a common epitope in the non-polymorphic amino acids of GRA6II peptide may explain this cross-reactivity. In order to verify this hypothesis, delimited versions of the GRA6II peptide were constructed, by truncation at the N terminus, that failed to improve the specificity of the original peptide, or deletion of the C-terminal residue, that eliminated false-positives, in mice (25).

**TABLE 3:** Amino acid sequence of GRA5, GRA6, and GRA7 synthetic peptides that better discriminate the different serotypes.

Peptide	Loci	Strain type	Amino acid sequence	References
GRA6II	GRA6	II	LHPGSVNEFDFLHPGSVNEFDFLHPGSVNEFDF	(28)
GRA6I/III	GRA6	I and III	LHPERVNVFDYLHPERVNVFDYLHPERVNVFDY	(28)
GRA7I	GRA7	I	LEQEVPESGEDGEDARQLEQEVPESGEDGEDARQLEQEVPESGEDGEDARQ	(29)
GRA7III	GRA7	III	PEHEVPESGEDREDARQPEHEVPESGEDREDARQPEHEVPESGEDREDARQ	(29)
Am6	GRA6	Atypical	GGNEGRGEGGGEDDRRALHPGSVNVFDY	(29)
Af6	GRA6	Atypical	GGNEGRGYRGRGEGGGEDDGRALHPERVNVFDY	(29)
Am7	GRA7	Atypical	AGEEPLTTSQNVNAGEEPLTTSQNVNAGEEPLTTSQNVN	(29)
6-I/III	GRA6	I and III	CLHPERVNVFDY	(25)
(d)6-I/III	GRA6	I and III	CLHPERVNVFD	(25)
6-II	GRA6	II	CLHPGSVNEFDF	(25)
(d)6-II	GRA6	II	CLHPGSVNEFD	(25)
7-II	GRA7	II	CVPESGKDGEDARQ	(25)
GRA5-Nt type I	GRA5	I	GSTRDVGSGGDDSEGARGREQQQVQQHEQNEDRSLFERGRAAVTGHPVRT	(26)
GRA5-Nt type II	GRA5	II	GSTCDTGSGGDDSEGAWGGEQQQVQQHGQSEDRSLFERGRAAVTGHPVRT	(26)
GRA5-Nt type III	GRA5	III	GSTRDVGSGADDSEGAGGRERQQVQQHEQNEDRSLFERGRAAVTGHPVRT	(26)
GRA6-Ct type I	GRA6	I	RRTGRRSPQEPSGDGGGNDAGNNAGNGGNEGRGYGGRGEGGAEDDRRPLHPERVNVFDY	(26)
GRA6-Ct type II	GRA6	II	RRTGRRSPQEPSGGGGGNDAGNNAGNGGNEGRG—-GEGG-EDDRRPLHPGSVNEFDF	(26)
GRA6-Ct type III	GRA6	III	RRTGRRSPPEPSGDGGGNDAGNNAGNGGNEGRGYGDRGEGGGEDDRAPLHPERVNVFDY	(26)
GRA7-III	GRA7	III	IKRTYRHFSPRKNRSRQPALEQEVPPSHEDGEDARQR	(39)
GRA7	GRA7	III	ESGEDREDAR	(33)
GRA6II/c	GRA6	II	CNNAGNGGNEGRG	(40)
GRA7III/d	GRA7	III	CGIKRTGGSGGGSGSGPAP- EHEVPESGEDR	(40)
GRA6 Africa 1/b	GRA6	Africa 1	CYRGRGEGG- GSGGGSGSGCYRGRGEG	(40)

Polymorphic amino acids are underlined.

Peyron et al. (26) proposed the use of recombinant polymorphic peptides of GRA5 and GRA6 antigens, specific for strains of *T. gondii* of genotypes I, II, and III (Table 3). The peptides were derived from the hydrophilic N-terminal region in native GRA5, the first 75 amino acids, and native GRA6 C-terminal region (172–230 amino acids). Derived peptides from GRA5 and GRA6 type 2 strains could differentiate genotype II strains from non-genotype II. Similar peptides derived from GRA5 and GRA6 genotypes I and III strains, failed to differentiate these two strains (30).

Sousa et al. (28) validated C-terminal GRA6-derived peptides for serotyping assay: peptide GRA6II, polymorphic specific for genotype II *Toxoplasma* strains, and the peptide GRA6I/III with polymorphisms specific for genotype I and III strains. Their findings were in accordance with genotyping results for 20 out of 23 cases associated with strains of the three main lineages. In addition, two peptides derived from the GRA7 locus with polymorphisms specific for genotype I (GRA7I) and genotype III (GRA7III) strains were investigated (29); peptide GRA7I provided only low sensitivity whereas peptide GRA7III appears to be informative for serotyping human infection with genotype III strains (29). A shorter version of the GRA7III C-terminal peptide was designed and specifically discriminates genotype III from non-genotype III infections in mice, rabbits, and humans (33). Xiao et al. (39) designed 12 peptides from GRA5, GRA6, and GRA7, specific for the archetypal genotypes I, II, and III. GRA5-derived peptides were from the N-terminal region and GRA6 and GRA7 from the C-terminus. One particular peptide, named GRA7-III distinguished genotype I from genotype III in infected mice with a specificity and sensitivity of 100% (39).

Xicotucal-Garcia et al. (37) designed new peptides of GRA6, GRA7, and SAG1 proteins. The peptides were designed with more SNPs, including linkers joining non-contiguous segments. Only with the GRA6 peptides it was possible to distinguish the three clonal genotypes.

## 5. Serotyping and non-archetypal genotypes

Non-archetypal strains, or atypical genotypes, exhibit, for the selected markers, a unique combination of alleles, discrete from the archetypal genotype, but also unique or different alleles. We hypothesize that infections with those atypical genotypes may induce a specific humoral response. Is it possible to provide serotyping assays for atypical genotypes using the polymorphic peptides defined so far, and based on archetypal strains? Mixed serum reactivity signatures, or reaction patterns exclusive of some non-archetypal strains, can be observed (28, 29, 38). Arranz-Solis et al. (38), using specific combinations of peptide ratios revealed unique profiles for the non-archetypal strains MAS (haplogroup 4), BOF (haplogroup 6), CAST (haplogroup 7), and Cougar (haplogroup 11). Furthermore, a distinct reactivity pattern using GRA6 derived peptides is able to distinguish haplogroup 11 and 12 from infections with archetypal North American/European strains (38).

Sequencing of loci GRA6 and GRA7 from 49 non-archetypal strains revealed 18 and 10 different amino acid sequences, respectively (29). Table 4 shows GRA6 polymorphisms of five non-archetypal strains, differing from clonal strains by one or two amino acids at the C-terminal region (SNP 223, 224, 227, and 230) (28). Three different sequences were described for the C-terminal region of those five atypical strains (Table 4). Strains GUY-2002-MAT and GUY-2002-KOE shared the same four SNPs as genotype I and genotype III strains, inducing the misclassification of the non-archetypal strains as genotype I/III. Strain GUY-2004-AKO differed from the peptide characterizing genotype I and III by a single amino acid. This single amino acid substitution was insufficient to distinguish *Toxoplasma* infections with strains harboring these alleles. Strains GUY-2003-BAS and GUY-2004-TER shared two SNPs with genotype II strains and two SNPs with genotype III strains. Strains that shared these polymorphisms displayed different reactivity profiles. The same atypical allele may induce a genotype II response, a genotype I/III response or a double response against both peptides.

**TABLE 4:** C-terminal amino acid polymorphisms for *GRA6* marker for genotype I (RH), genotype II (Beverley), genotype III (NED), and five non-archetypal strains (28).

Strain	223	224	227	230
RH	E	R	V	Y
BEVERLEY	G	S	E	F
NED	.	.	.	.
GUY-2003-BAS	G	S	.	.
GUY-2004-TER	G	S	.	.
GUY-2002-MAT	.	.	.	.
GUY-2002-KOE	.	.	.	.
GUY-2004-AKO	.	H	.	.

Periods (.) indicate amino acids identical to the sequence of RH strain.

Three peptides specific for some non-archetypal strains were selected from GRA6 and GRA7 antigens (29). From GRA7, peptide (Am7) contained polymorphisms specific for three haplotypes found in South America. Two other peptides were derived from GRA6, one specific for South American strains (peptide Am6) and the other specific for African strains (peptide Af6). These two peptides were selected from the most representative haplotypes among strains from Africa and South America (29). Peptides Am6 and Af6 had low specificities, since they react with sera from cases with strains belonging to the three archetypal lineages. Peptide Am7 was specific but poorly sensitive.

Another peptide from GRA6 locus, named GRA6 Africa 1/b was described for Africa 1 lineage (40). Peptide GRA6 Africa 1/b is derived from an insertion sequences found in genotype III and Africa 1 genotype. This epitope differs between genotype III and Africa 1 at position 206 involving a glycine to arginine substitution. This peptide had a sensitivity of 100% for Africa 1/b genotype, but cross-reacted with genotype II and III (40).

## 6. Serological signature, geographic origin, and disease outcome

The serotype II profile is significantly more frequent in sera from Europeans, independent of clinical presentation of the disease (Table 5) (26, 28, 30, 40–43). In USA, serotype II was also associated with congenital toxoplasmosis (43). In contrast, serotypes I/III are more prevalent in South America, where non-archetypal genotypes prevail (26, 28, 30, 37, 44, 45). Few serotyping studies have been conducted in sera of African origin. However, as in South America, serotype I/III was also more prevalent than serotype II (28). Serotype I was associated with retinal lesions in patients from Colombia (45), and was the most frequent among congenital infections in Mexico (37).

**TABLE 5:** *Toxoplasma gondii* serotypes according to pathology and geographic origin.

Pathology	Geographic origin	Sample size	Prevalent serotype (%)						References
			I (%)	II (%)	III (%)	I/III (%)	AT* (%)	NR (%)	
Congenital	Poland	28		71.4			14.3	14.3	(41)
	Portugal/France	44		65.9		11,4		22.7	(28)
	Mexico	30	53		13	2		30	(37)
	USA	183		36		15	49		(43)
Ocular	Denmark	1		100					(42)
	Germany	114		41		7	8	44	(46)
	Brazil	22				59	27	14	(47)
	Colombia	23	43	9				43	(45)
Multivisceral	French Guiana	4		25		50		25	(28)
Immunosuppressed	Portugal/France	31		58		7	10	26	(28)
	Africa	8				63	25	13	(28)
Immunocompetent asymptomatic	Portugal/France	130		43		10	8	39	(28)
	Africa	80		11		29	14	46	(28)
	South America	148		3		46	18	32	(28)
	Colombia	20	50		10			40	(45)

| *Atypical serotype (AT) was considered for serum samples that cross-reacted with genotype II and genotype I/III peptides.

Atypical serologic signatures with different reactivity against GRA6 and GRA7 type II and I/III were identified among Germans with ocular toxoplasmosis (46). Three of these atypical profiles had been identified in sera associated with a waterborne outbreak of human toxoplasmosis in Brazil (47). In addition, a novel non-reactive (NR) serotype was identified among *Toxoplasma*-infected uveitis cases in Germany who developed ocular toxoplasmosis (46). Non-archetypal strains typically possess entirely divergent polymorphic epitopes at GRA6 or GRA7, and these epitopes do not necessarily elicit antibodies capable of reacting with the diagnostic allelic peptide motifs. Alternatively, it is also possible that host genetic factors preclude the ability of ocular toxoplasmosis patients to produce allele-specific antibodies at GRA6 and GRA7 or that the polymorphic epitopes are not immunogenic in this patient cohort (48, 49). NR serotype patients with active ocular toxoplasmosis who have a higher risk of recurrences can be expected to receive appropriate management including preventive treatment.

## 7. Limitations, the future, and conclusion

*Toxoplasma* serotypes are defined by the serological profile based on the antibodies reaction to genotype-specific peptides. The peptides described thus far have limited utility. Those peptides only distinguished genotype II strains from non-genotype II. As a consequence, it is not possible to differentiate the clonal genotype III, and all the non-archetypal genotypes, as well as genotype I. The limited number of loci that have been investigated in depth raised the same problems found in single locus genotyping (28, 29). This is especially true in infections with non-archetypal strains (28). The epitope expressed by the infecting strain ultimately determined the reactivity against the diagnostic peptides, not the genotype of the infecting strain (47). For each lineage, peptides must be selected from as a high a number of antigens as possible. Therefore, in order to validate serotyping as an expedient method for genotyping and diagnosis, several peptides from different strain-specific markers need to be characterized (29).

In addition to specificity problems, the sensitivity of serotyping is a limitation given that some infections cannot presently be serotyped. Serotyping with a larger number of peptides, specific for both archetypal and non-archetypal strains can be anticipated to enhance the sensitivity of the method and consequently diminish the number of non-typed samples (29). For congenital toxoplasmosis and other *T. gondii*-related diseases (cerebral, pulmonary, and ocular) from Europe, the number of non-serotyped infections was 22.7 and 33.3%, respectively (28). Similar results were obtained for congenital infections from Poland (14.3%) by Nowakowska et al. (41). In addition, for immunocompetent asymptomatic cases from Europe and Africa serotyped with GRA6 archetypal peptides, 39.2 and 46.3%, respectively of the sera could not be serotyped (28). These non-reactive sera were later defined as a new serotype (46). Nevertheless, serotyping has clear potential in diagnosis. Given that it is non-invasive, serotyping can be readily deployed in large-scale epidemiological studies (32) with the objective of defining serotype distribution among the continents and in wild, feral, and domesticated hosts to understand whether strain selection is dependent on the host species. Regarding human infection, serotyping will enhance our understanding of the relationships between strain genotype and pathogenesis (28, 29, 46). Accomplishing these objectives will positively influence the diagnostic performance and treatment of the cases. Serotyping may be useful in the detection of a re-infection with a different strain, which may be particularly relevant in the follow-up of pregnant women. Last, since toxoplasmosis is a foodborne disease, serotyping can play a critical role for detection of *T. gondii* infections by non-archetypal strains in livestock and meat products destined for human consumption.

This review discusses the state of the art of the serological typing of *T. gondii*, known as serotyping. Many questions remain regarding host-parasite interaction, parasite biology and virulence factors. Understanding the role of strain-specific *Toxoplasma* antigens on infection and consequent host immune response underpins the selection of the best targets for *in vitro* genotype detection. Multilocus genotyping of *T. gondii* has revealed a markedly high genetic diversity. This population diversity is challenging to a serotyping approach, particularly for not clonal lineages. The many peptides suggested for genotype-focused serotyping, in tandem with the few only sensitive and specific peptides that have been reported reflects this difficulty. However, for clonal lineages, serotyping may become a routine. Selection of efficient markers is time and cost expensive, and there is still a long way to go. Nevertheless, we consider that this typing method offer promising advantages and a similar multilocus approach must be followed with peptides from different antigens. Indeed, we ourselves continue to investigate novel peptides for serotyping type II strains.

## Author contributions

SS and MF wrote the manuscript. JC proposed the subject and was responsible for the critical review of the manuscript. All authors read and approved the final manuscript.
